# Effect of Samarium on the Microstructure and Corrosion Resistance of AZ91 Magnesium Alloy Treated by Ultrasonic Vibration

**DOI:** 10.3390/ma11112331

**Published:** 2018-11-20

**Authors:** Yang Chen, Zheng Yin, Hong Yan, Guo-Hua Zhou, Xiao-Quan Wu, Zhi Hu

**Affiliations:** 1 Institute of Advanced Forming, Nanchang University, Nanchang 330031, China; Chenyang_bf@163.com (Y.C.); Zhenyin1114@163.com (Z.Y.); yanhong_wh@163.com (H.Y.); yhong_177@sina.com (X.-Q.W.); 2 Key Laboratory of Light Alloy Preparation & Processing in Nanchang City, Nanchang 330031, China; 3 Physical Science and Technology College, Yichun University, Yichun 336000, China; zgh7605@163.com

**Keywords:** AZ91 magnesium alloy, Sm, microstructure, corrosion resistance, ultrasonic vibration

## Abstract

The effects of samarium (Sm) on the microstructure and corrosion behavior of AZ91 magnesium alloy treated by ultrasonic vibration were investigated by scanning electron microscopy, X-ray diffraction, transmission electron microscopy, and electrochemical measurements. The results showed that the addition of Sm resulted in the formation of Al_2_Sm, which reduced the volume fraction of the β-Mg_17_Al_12_ phase and changed its morphology to fine granular. The AZ91–Sm alloys treated by ultrasonic vibration revealed relatively lower weight loss, hydrogen evolution, and corrosion current density values compared to the ultrasonic-treated AZ91 alloy prepared without Sm. Locally, a coarse β phase in the ultrasonic-treated AZ91 alloy accelerated the possibility of micro-galvanic corrosion growing into the matrix. In the prepared AZ91–Sm alloys treated by ultrasonic vibration, the fine β and Al_2_Sm phases reduced the probability of micro-galvanic corrosion growth and, therefore, formed a uniform corrosion layer on the surface of the alloys.

## 1. Introduction

As the lightest metallic materials, magnesium (Mg) and its alloys are useful materials for the aerospace, automobile, and electronic industries [1,2]. Many magnesium alloys are also expected to be used as biodegradable materials for medical applications [1,2]. Mg and its alloys present many excellent properties, such as machinability, castability, biocompatibility, and anti-electromagnetic radiation properties [3]. In particular, AZ91 alloys are widely used because of their excellent mechanical properties, including high damping characteristics, excellent electromagnetic shielding, and recyclability [4,5]. Nonetheless, corrosion remains a particular concern for Mg and its alloys because of their active chemical properties and the low equilibrium voltage of Mg, resulting in poor corrosion resistance [6,7,8].

In recent years, researchers have found that the addition of rare earth elements can purify the melt, refine the microstructure, and strengthen properties such as the strength or hardness at room or elevated temperatures and the corrosion resistance, of Mg alloys [6,7]. The AE (Mg-Al-RE) series of alloys is based on the addition of rare earth elements. These materials exhibit enhanced creep resistance due to the complete inhibition of Mg_17_Al_12_ intermetallic compound and the formation of highly stable Al–RE phases, such as Al_2_RE, Al_3_RE, or other RE phases [9]. The RE-rich phase, including Al_2_Yb [10], Al_2_Y [11], Al_11_Nd_3_ [12], and Al_3_Er, plays a critical role in the improved corrosion resistance of magnesium alloys [3]. Wu et al. [13], for example, found that the addition of samarium (Sm) to AZ92 alloy decreased the volume fraction of the β-Mg_17_Al_12_ phase from 0.29% to 0.075% as the content of Sm increased. The corrosion rate of the AZ92 alloy with the addition of 0.5 wt % of Sm decreased by 54% compared to that of the matrix alone. Overall, Sm, with the highest atomic number among the light rare earth elements, is one of the most effective RE elements for the refinement of intermetallic compounds and the improvement of the corrosion resistance of magnesium alloys [14].

In addition to the use of RE elements as intermetallic refiners in Mg alloys, physical methods such as ultrasonic vibration can also be used to refine the grain size during solidification [15]. Many studies have demonstrated that ultrasonic vibration treatment of alloys can effectively enhance ductility [16], elongation [17], tensile strength [18], and hardness [19], with refinement of the second phase and improved microstructure. Zhang et al. [20,21] investigated the effects of high-intensity ultrasonic vibration on the morphology and mechanical properties of an Mg–Al binary alloy and observed that the ultrasonic treatment had a great impact on the size and microstructure of the β-Mg_17_Al_12_ phase. The β-Mg_17_Al_12_ phase became finer, more homogeneous, and adopted a discontinuous network shape. Although the mechanical properties of ultrasonic-treated magnesium alloys have been intensively investigated, little attention has been devoted to the corrosion resistance of these treated magnesium alloys. Thus, the goal of this work was to investigate the influence of Sm on the microstructure evolution and corrosion behavior of AZ91 magnesium alloys treated with ultrasonic vibration by determining the morphology, size, and distribution of the second phase in these alloys.

## 2. Experimental

### 2.1. Material Preparation and Microstructural Observation

A commercial AZ91 magnesium alloy ingot was selected as the experimental raw material. The rare earth element Sm was introduced into the Mg–Sm (30 wt %) master alloy at concentrations of 0, 0.5, 1, 1.5 wt %. The AZ91 magnesium alloy and the Mg–Sm master alloy were prepared by melting in a steel crucible inside a resistance furnace under the protection of SF_6_ and CO_2_ (VSF_6_/VCO_2_ = 1:6) atmosphere. The melted alloys were then treated by high-energy ultrasonic vibration for 10 min at 750 °C. The typical experimental setup for the ultrasonic vibration technique has been previously described in the literature [3]. The power of the ultrasonic generator was 0.6 KW, and the frequency was 20 KHz. Finally, the melt was poured into metallic molds with dimensions of 150 mm in length and 15 mm in diameter. After the preparation, the actual components of each alloy were measured by inductively coupled plasma emission spectrometry (ICP–AES, PerkinElmer, Norwalk, CA, USA), and the results are listed in Table 1. In order to observe the microstructure of the prepared AZ91–xSm magnesium alloys by scanning electron microscopy (SEM, JEOL Ltd., Tokyo, Japan) with energy dispersive spectroscopy (EDS), the specimens were polished with 400^#^–2000^#^ SiC paper and 5–0.5 μm diamond paste on the MP-2A metallographic polishing machine, and the specimen surface was etched with 4 vol % mixed solution of nitric acid and ethanol for 10 s. The intermetallic compound was identified by X-ray diffraction (XRD, Bruker, Karlsruhe, Germany) and transmission electron microscopy (TEM, JEOL Ltd., Tokyo, Japan). A specimen with a 3 mm diameter and 70 μm thicknesses was prepared by sandpaper polishing for TEM observation. The specimen was cut and embedded into resin, then ground with 2500-grit SiC paper and polished by diamond paste for XRD observation.

### 2.2. Immersion Testing

**Figure 1:**
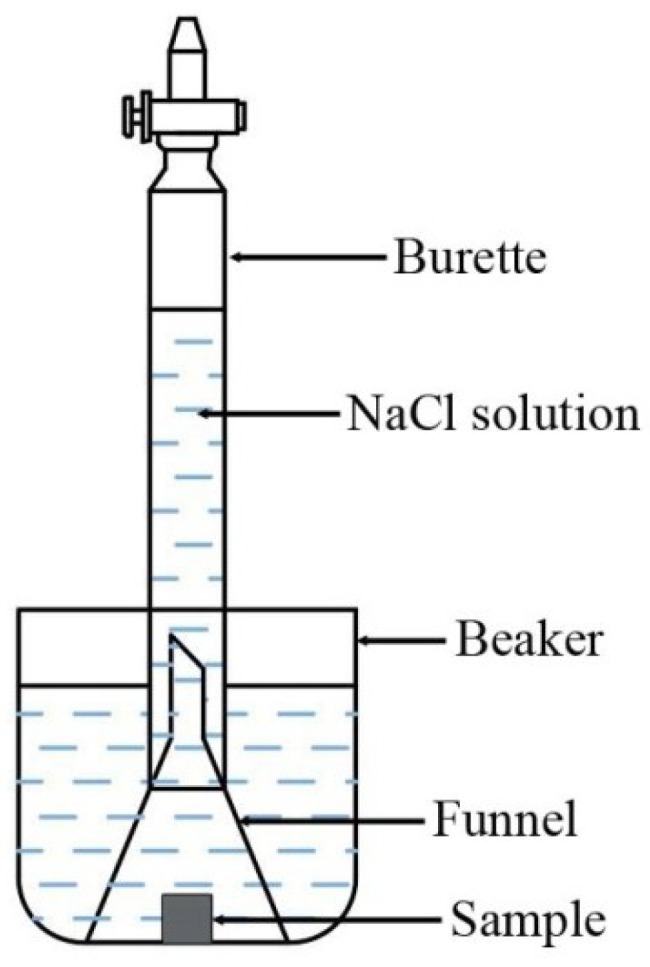
Schematic of corrosion test equipment.

The corrosion rate was evaluated by measuring (1) the amount of hydrogen that evolved during corrosion in an aqueous solution of 3.5 wt % NaCl and (2) the weight loss of the samples. The test samples were cut into 10 × 10 × 10 mm squares by wire cutting, then the surface of each sample was wet-ground to a 1200-grit finish using abrasive paper, cleansed with distilled water, and dried in a compressed hot air flow. All samples were weighed using an electronic scale with an accuracy of 0.001 g and then immersed into the NaCl solution for 24 h. After immersion testing, the corroded specimens were cleaned with distilled water and dried. They were then immersed in an aqueous solution (200 g/L CrO_3_ + 10 g/L AgNO_3_) for 5–10 min to remove the corrosion products. The specimens were quickly washed with distilled water, dried with a cool flow wind, and weighed again. To measure the hydrogen evolution reaction, we recorded the starting value and the final value. The experimental setup for this study is shown in Figure 1. The corrosion rate was calculated using Equation (1):

(1) $$V=\frac{∆M}{A\times t}$$

where V (mg·cm^−2^·day^−1^ or mL·cm^−2^·day^−1^) represents the corrosion rate, Δ*M* (mg or mL) is the weight difference or the volume difference during the immersion test, *A* (cm^2^) is the total area of the specimen, and *t* (days) is the corrosion time. All the experiments were carried out at room temperature and repeated five times for good reproducibility. We calculated the average of five experiments.

### 2.3. Electrochemical Testing

Electrochemical testing of each alloy was performed using a three-electrode flat cell configuration with a platinum mesh as the counter electrode, a saturated calomel as the reference electrode, and the specimen as the working electrode. To prepare the specimens for testing, the samples were cut into cubes with dimensions of 10 mm × 10 mm × 1 mm and ground with 1000-grit SiC paper. The electrolyte was 3.5 wt % NaCl solution in a volume of 400 mL. Polarization measurements were carried out using the electrochemical workstation Princeton P4000, with an initial scan rate of 2 mV/s and a potential range of ±350 mV versus the open circuit potential.

## 3. Results

### 3.1. Microstructure

**Figure 2:**
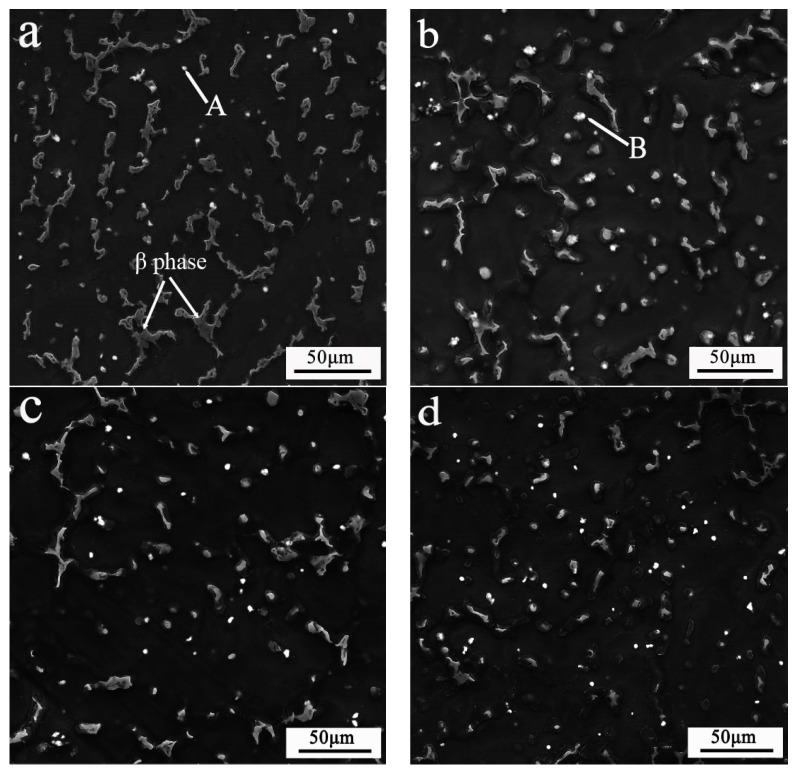
SEM images of AZ91–xSm magnesium alloys treated by ultrasonic vibration, where x = (**a**) 0 wt %; (**b**) 0.5 wt %; (**c**) 1 wt %; (**d**) 1.5 wt %.

**Figure 3:**
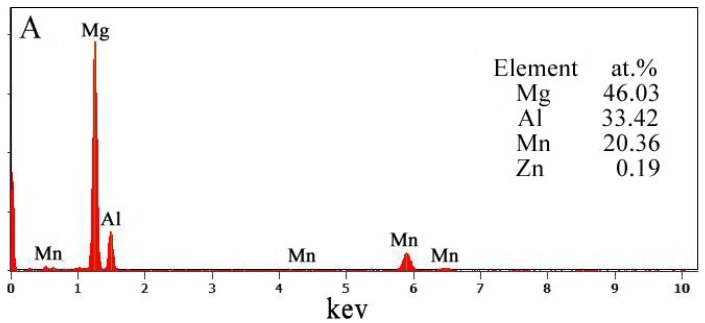

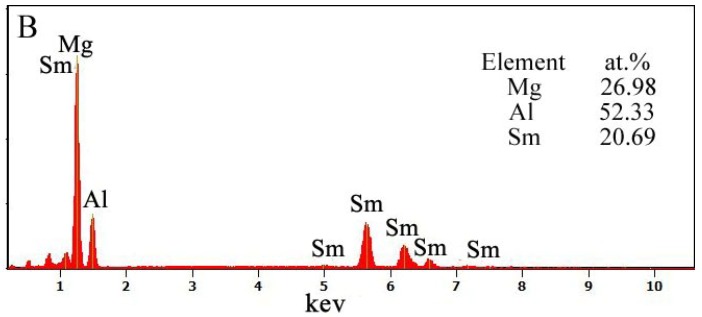
Energy dispersive spectroscopy (EDS) images: (**A**) the Al-Mn phase in AZ91 and (**B**) the Sm-rich in AZ91-0.5Sm magnesium alloy.

The initial microstructures of the alloys treated by ultrasonic vibration are shown in Figure 2a, which also shows that the primary phase (α-Mg) was separated by the relatively coarse β-Mg_17_Al_12_ phase. Figure 2b–d show the microstructure of AZ91–xSm (x = 0.5, 1, 1.5 wt %) alloys. The size of the β-Mg_17_Al_12_ phase was obviously smaller. With increasing Sm amounts, the β-Mg_17_Al_12_ phase became more homogeneous and distributed in granular form in the alloy, becoming distinct at 1.5% Sm. We can also observe some light white particles in these images. For further verification, we used EDS to detect the composition of these materials. Figure 3 shows the EDS of the areas indicated by arrows A and B in Figure 2a,b. We can see that the granular particles in Figure 2a (indicated by arrow A) were made of Al–Mn intermetallic compound. Similarly, the Al–Mn intermetallic compound in the AZ61 magnesium alloy was characterized by Al_8_Mn_5_ [4], whereas the area indicated by arrow B in Figure 2b was the Sm-rich phase.

**Figure 4:**
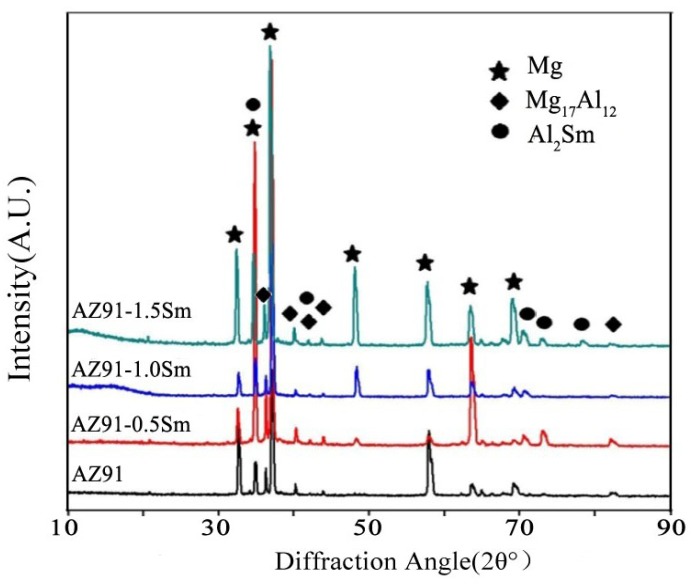
XRD analysis of AZ91–xSm (x = 0, 0.5, 1.0, 1.5 wt %) magnesium alloys with ultrasonic treatment.

**Figure 5:**
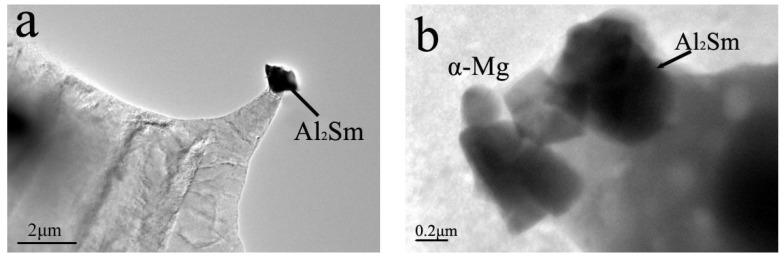

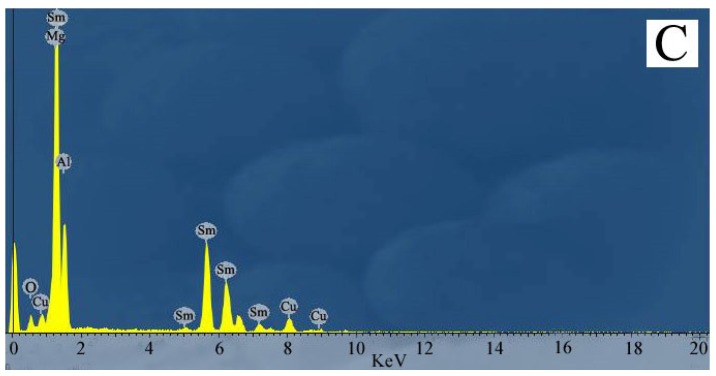
TEM image of ultrasonic-treated AZ91–xSm (x = 0, 0.5, 1.0, 1.5 wt %) magnesium alloys; (**a**,**b**) Al_2_Sm particle; (**c**) EDX results.

We next performed XRD analysis of these AZ91–xSm materials (Figure 4) and found that the peaks corresponding to the five-pointed star and diamond represented α-Mg and β-Mg_17_Al_12_, respectively. When a small amount of Sm was added, a peak represented by a circle was observed. The greater the Sm addition, the higher this peak value was. TEM (Figure 5a,c) and XRD results could clearly classify the Sm-rich phase as the phase of Al_2_Sm. In our previous study, the similar Sm-rich phase in Mg–6Al–0.8Zn–2.0Sm alloys was classified as Al_2_Sm phase by its diffraction patterns. Therefore, the peak represented by a circle is the Al_2_Sm phase.

**Figure 6:**
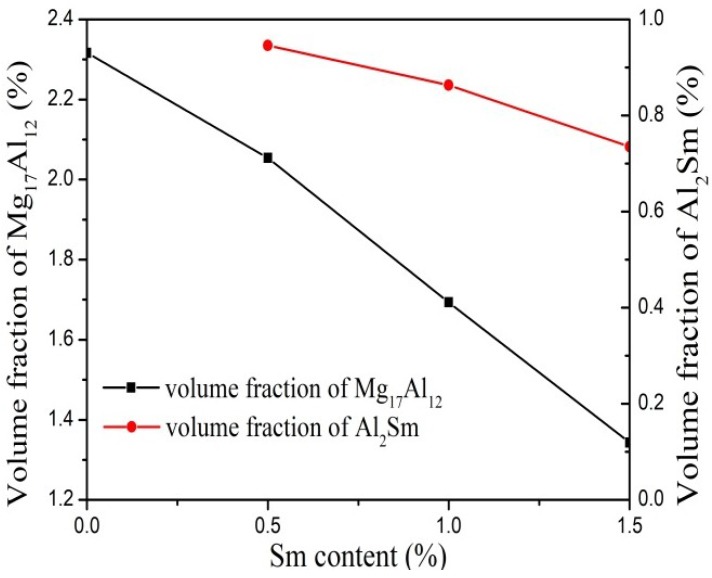
The volume fraction of β-Mg_17_Al_12_ and Al_2_Sm particles in ultrasonic-treated AZ91–xSm (x = 0, 0.5, 1.0, 1.5 wt %) magnesium alloys.

Figure 6 shows the volume fraction of Al_2_Sm particles and β-Mg_17_Al_12_ of the ultrasound-treated alloy measured by the Image-Pro Plus 6.0 image analyzer (IPP) (Media Cybernetics, Sarasota, FL, USA). The volume fraction of β-Mg_17_Al_12_ showed a sharp decline from 2.3% to 1.3% as the amount of Sm increased from 0 to 1.5%. The main reason for this change is that the formation of Al_2_Sm consumes a large amount of Al element, causing a decrease in the volume fraction of the β phase. Also the ultrasonic treatment can affect the volume fraction of the β phase. Sun et al. [22] reported a decrease in the volume fraction of the β phase from 9.4% to 6.6% after ultrasonic vibration. This effect is due to the breakdown of the dendritic structure by ultrasonic treatment. At the same time, in our study, the volume fraction of Al_2_Sm particles decreased from 0.95% to 0.74%. This is because many Al_2_Sm particles with size of ~200 nm dispersed in the matrix were too small to measure, as shown in Figure 5b, resulting in an apparent decrease in the Al_2_Sm particle volume fraction. Previous research [23] revealed that the maximum pressure caused by transient cavitation was far greater than the shear strength of the Al_2_Sm phase, causing the coarse petal-shaped Al_2_Sm phase to break apart from the roots of the petals and form finer polygonal particles in the alloy melts. The volume fraction and average diameter of the Al_2_Sm phase in the alloy after ultrasound treatment were obviously decreased.

### 3.2. Weight Loss and Hydrogen Evolution

**Figure 7:**
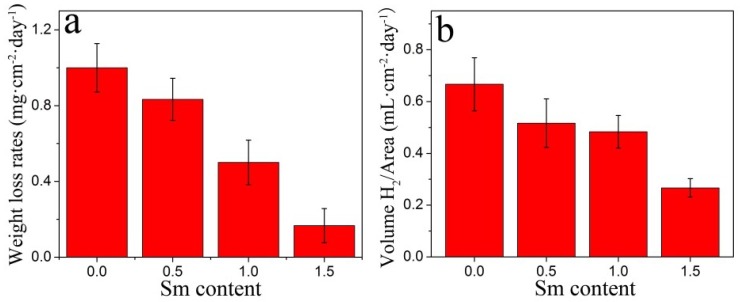
The corrosion rate of AZ91–xSm (x = 0, 0.5, 1.0, 1.5 wt %) magnesium alloys treated with ultrasound, as measured by (**a**) weight loss (**b**) hydrogen evolution reaction.

Figure 7a,b display the histogram of weight loss and hydrogen evolution rates of the ultrasonic AZ91–xSm alloys immersed in 3.5 wt % NaCl aqueous solution for 1 day. In addition, for alloys with Sm (0.5, 1.0, and 1.5 wt %), their corrosion rates were obviously lower than those of alloys without Sm. Strikingly, the alloy prepared with 1.5 wt % Sm presented the lowest corrosion rate, with weight loss of about 0.167 mg·cm^−2^·day^−1^ and hydrogen evolution of 0.247 mL·cm^–2^·day^–1^, respectively. These rates were decreased by 83.3% and 60.0%, respectively, compared to the rates for AZ91 lacking Sm, with weight loss of about 1 mg·cm^–2^·day^–1^ and hydrogen evolution of 0.667 mL·cm^–2^·day^–1^. This indicated that the Sm modification effectively promoted corrosion resistance in the magnesium alloys, and, as the Sm addition increased, the corrosion resistance of the alloys improved significantly.

### 3.3. Potentiodynamic Polarization

**Figure 8:**
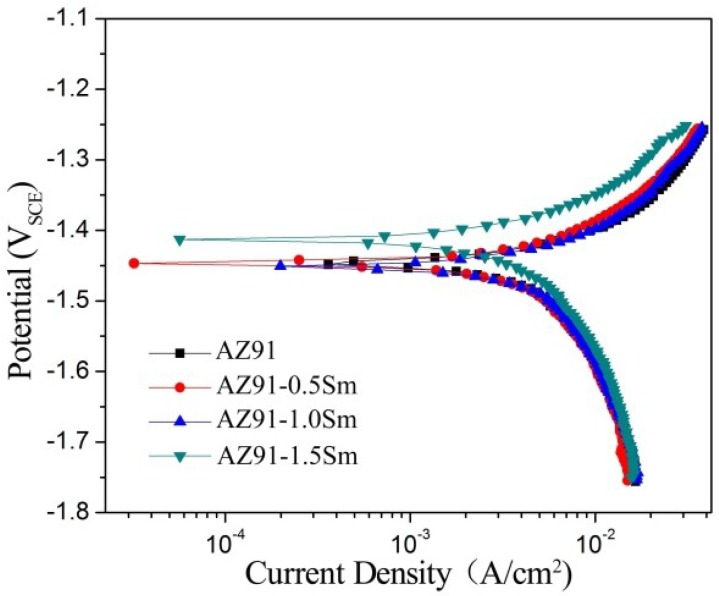
Tafel curves of AZ91–xSm (x = 0, 0.5, 1.0, 1.5 wt %) magnesium alloys treated with ultrasound.

Figure 8 shows the potentiodynamic polarization curves of the ultrasonic AZ91–xSm magnesium alloys in the 3.5 wt % NaCl aqueous solution. The corrosion potentials (*E_corr_*), corrosion current density (*I_corr_)*, anode Tafel slopes (*β_a_*), and cathode Tafel slopes (*β_c_*) of each alloy were fitted by VersaStudio software (P4000, Princeton, NJ, USA), and the polarization resistance (*R_p_*) of each sample was calculated by Equation (2) [24,25]. The results are listed in Table 2. It can be observed that the addition of Sm made the *I_corr_* lower than that of the base alloy, and the *I_corr_* decreased with the increase of Sm. The alloy prepared with Sm at 1.5 wt % had the lowest *I_corr_* (16.6 mA/cm^2^), corresponding to only 57.5% of the value for the base alloy, and *E_corr_* was the highest for this material. According to the anode and cathode Tafel slopes, *β_c_* was much larger than *β_a_*, indicating that the corrosion process was controlled by the cathode reaction. The *R_p_* values varied from 3.8 to 7.5 Ω·cm^2^ when 0–1.5 wt % Sm was added to the AZ91 alloy. The increased *R_p_* values for the potentiodynamic fitting data confirmed the inhibition of the cathodic reaction. These results are in accordance with those of the immersion test, in which Sm addition improved the corrosion resistance of the AZ91 alloy.

(2) $$R_{p}=\frac{\beta_{a}\beta_{c}}{2.3\left(\beta_{a}+\beta_{c}\right)I_{corr}}$$

### 3.4. Corrosion Morphology

**Figure 9:**
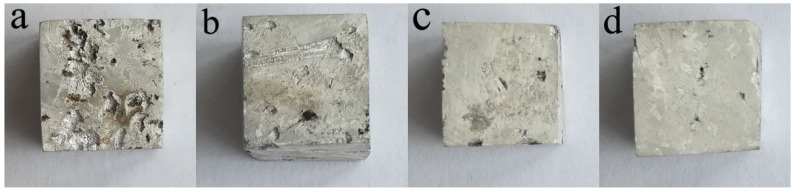
Macroscopic corrosion morphology of AZ91–xSm magnesium alloys treated with ultrasound, where x = (**a**) 0 wt %; (**b**) 0.5 wt %; (**c**) 1 wt %; (**d**) 1.5 wt %.

Figure 9 shows the macroscopic corrosion morphology of the AZ91–xSm in 3.5 wt % NaCl aqueous solution for 24 h and the removal of the corrosion products. As can be seen from the figure, all samples exhibited varying degrees of surface corrosion with some relatively deep corrosion pits. In general, the degree of surface corrosion decreased with the increase of Sm content. The addition of 1.5 wt % Sm considerably decreased the surface degradation of the specimens: the size and number of corrosion pits on the surface dramatically decreased, and the corrosion degree was the lowest. This result is consistent with the findings of the electrochemical test.

**Figure 10:**
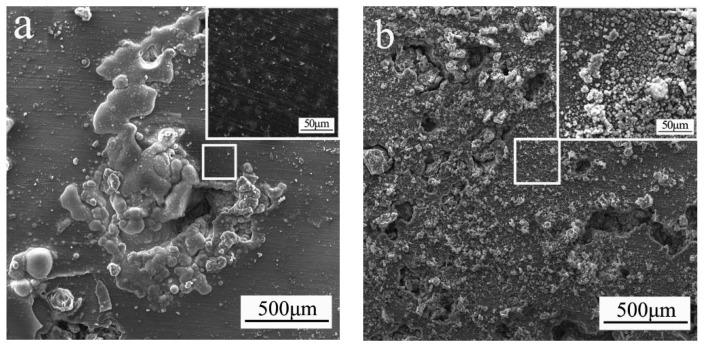
Morphology of corrosion products of the ultrasonic-treated (**a**) AZ91 alloy and (**b**) AZ91–1.5Sm alloy after immersion in aqueous 3.5 wt % NaCl solution for 24 h.

The microscopic corrosion morphology of the AZ91 and AZ91–1.5Sm materials in aqueous 3.5 wt % NaCl solution for 24 h was determined, and the results are shown in Figure 10. The surface of the alloy lacking Sm exhibited a large corrosion pit, as illustrated in Figure 10a, and had many corrosion products and cracks. The higher magnification image reveals that other areas hardly presented corrosion product layer, indicating that these sites did not undergo severe corrosion, that is, the corrosion occurred only locally. However, the surface of the alloy material prepared with 1.5 wt % Sm, shown in Figure 10b, shows fewer corrosion pits of smaller diameter. Additionally, most areas were covered with a uniform layer of corrosion products (a high-magnification image of a local area is shown in the inset at the upper right).

**Figure 11:**
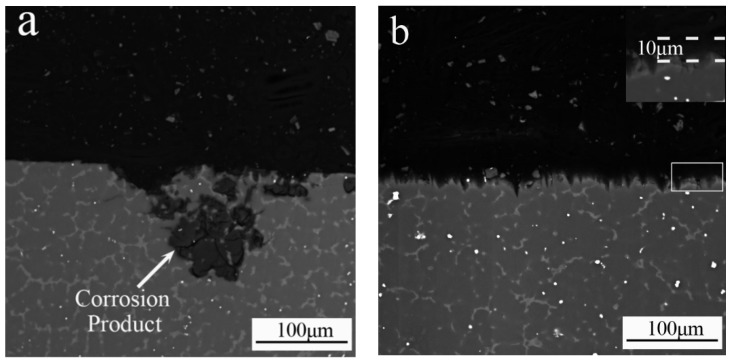
Back-scattered electron imaging of a corrosion longitudinal section of the ultrasonic-treated (**a**) AZ91 alloy and (**b**) AZ91–1.5Sm alloy after immersion in aqueous 3.5 wt % NaCl solution for 1 h.

SEM imaging was performed of the corrosion longitudinal sections of AZ91 and AZ91–1.5Sm alloys after immersion in 3.5 wt % NaCl aqueous solution for 1 h, as shown in Figure 11. The black part is the epoxy resin, and the gray part is the alloy structure. The corrosion morphology is consistent with the above findings and indicates that the ultrasonic-treated AZ91 magnesium alloy presented a smaller area of corrosion but formed deeper and larger corrosion pits (~120 μm) than the ultrasonic-treated AZ91–1.5Sm, which presented a larger area of corrosion but with shallow and small pits (10–20 μm), presenting a serrated structure. As shown in the high-magnification image of Figure 11b, we can see a uniform corrosion product layer with a thickness of 10 μm on the surface of the alloy. Compared to the base alloy, the modified alloy exhibited a homogeneous general corrosion, and the degree of corrosion was evidently smaller. These morphological findings are in agreement with the results of the immersion and electrochemical tests.

## 4. Discussion

The microstructure of intermetallic compounds can be significantly influenced by ultrasonic vibration [26]. Whefn ultrasound was applied to the AZ91 magnesium alloy, the coarse dendritic microstructure gradually transformed into a globular structure and showed a significant reduction in size [27]. Zhong et al. [28] reported that the long needle-like β-Al_5_FeSi phase evolved to a refined acicular one, and its size decreased from 200 μm to 80 μm after application of ultrasonic vibration. The intermetallic compound in the 6% Si alloy showed an average equivalent diameter of 77 μm, while the average equivalent diameter in the 12% Si alloy was 59 μm (initially coarse particles with a length of 10 mm) after the application of ultrasonic vibration [29]. The acoustic cavitation induced by ultrasonic vibration is the main cause of the decrease in the size of the intermetallic compound and of the more even distribution. During cavitation, bubble growth and local high temperature and pressure occur, resulting in a decrease in the local temperature around the cavitation bubble. Under the action of sound waves, the cavitation bubbles break up to produce strong shock waves that can break the growing crystals to achieve a refined second phase [30]. In our study, we clearly observed the apparent refinement and uniform distribution of the second phase in the AZ91–1.5Sm sample (Figure 2d). Additionally, the coarse network-like β-Mg_17_Al_12_ phase changed into a rod-like or granular phase.

**Figure 12:**
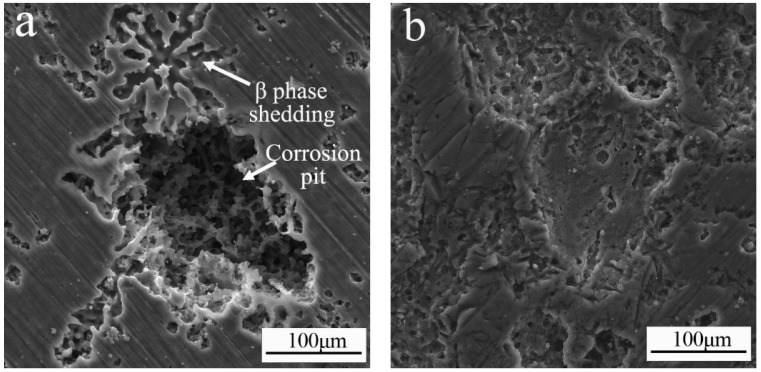
Corrosion morphology of the ultrasonic-treated (**a**) AZ91 alloy and (**b**) AZ91–1.5Sm alloy after immersion in aqueous 3.5 wt % NaCl solution for 24 h.

The refinement of intermetallic compounds has a significant effect on the corrosion resistance of magnesium alloys [31,32]. Chen et al. [33] deduced that the β phase exerts a “size effect” on the AZ91 Mg alloy: the large island-like β phase became finer and more evenly distributed, and the *I_corr_* values of AZ91 decreased by at least two orders of magnitude to enhance corrosion resistance. In this study, the analysis of the morphology of the ultrasonic-treated AZ91 surface indicated local corrosion, as shown in Figure 12a. After immersion in NaCl aqueous solution for 24 h, corrosion occurred in the β-Mg_17_Al_12_ phase and spread out along it, forming large corrosion pits. After the addition of 1.5 wt % Sm, as illustrated in Figure 12b, the surface of the alloy was slightly corroded and exhibited overall corrosion.

**Figure 13:**
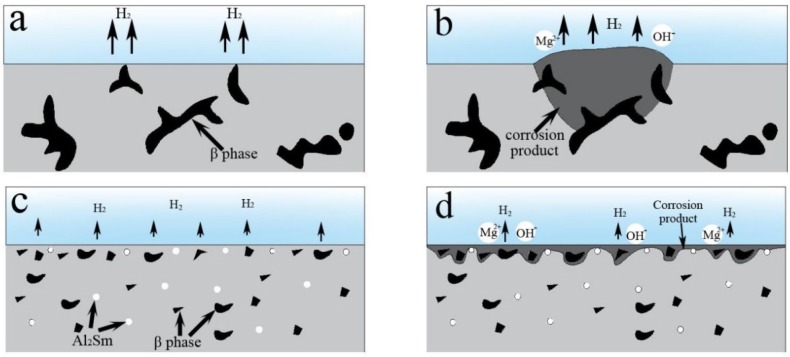
Schematic illustrations of the ultrasonic-treated (**a**,**b**) AZ91 alloy and (**c**,**d**) AZ91–1.5Sm alloy during the corrosion process.

A schematic diagram of the corrosion process of the ultrasonic-treated AZ91 and AZ91–1.5Sm alloys is shown in Figure 13. In the initial stage of corrosion (illustrated in Figure 13a), the AZ91 alloy is composed of only a coarse network β phase and the matrix. After immersion in aqueous NaCl solution (Figure 13b), the β phase acts as a cathode with respect to the matrix and Mg^2+^ ions, and OH^−^ ions form at the alloy surface because of micro-galvanic corrosion between the coarse β phase and the matrix. These processes are described by reactions (3) and (4) [34]:

Mg → Mg^2+^ + 2e^−^ (3)

2H_2_O + 2e^−^ → H_2_ + 2OH^−^ (4)

Large corrosion products began to appear in local areas of the alloy surface, and the corrosion product had a tendency to grow into the interior of the alloy along the coarse β phase, consistent with Figure 11a.

After addition of 1.5% Sm to the AZ91 alloy (as shown in Figure 13c), the coarse network β phase transformed into small, dispersed particles. At the same time, bright white Al_2_Sm particles appeared. After NaCl solution immersion (Figure 13d), the Al_2_Sm phase and the β phase acted as cathodic phases, accelerating micro-galvanic corrosion in the matrix. Because of the very small second phase, the micro-galvanic corrosion was unable to grow into the interior of the alloy and continue to react in other surface areas of the alloy. With further progress of the micro-galvanic corrosion, the entire surface of the AZ91–1.5Sm alloy formed a uniform corrosion layer (shown also in Figure 11b).

## 5. Conclusions

1. The addition of Sm to the AZ91 alloy treated by ultrasonic vibration resulted in the formation of an Al_2_Sm phase about 2 μm in size, which reduced the volume fraction of the β-Mg_17_Al_12_ phase and changed the morphology of β-Mg_17_Al_12_ phase to fine granular.
2. The weight loss, 0.167 mg·cm^−2^·day^−1^, and hydrogen evolution, 0.247 mL·cm^−2^·day^−1^, revealed that the ultrasonic AZ91–1.5Sm alloy was characterized by a relatively lower corrosion rate than the alloy prepared without Sm addition. Additionally, the electrochemical test also showed increased corrosion resistance of alloy specimens with increased Sm content.
3. The size and morphology of the second phase had a key role in the micro-galvanic corrosion growth process. The locally coarse β phase accelerated the possibility of micro-galvanic corrosion growth into the matrix. The fine β and Al_2_Sm phases in the alloy reduced the probability of micro-galvanic corrosion growth, resulting in the formation of a uniform corrosion layer.

## Figures and Tables

**Table 1:** Composition (wt %) of AZ91–xSm magnesium alloys treated with ultrasonic vibration.

Alloy	Al	Mn	Zn	Sm	Mg
AZ91	9.23	0.29	0.67	---	Bal.
AZ91–0.5Sm	9.04	0.35	0.82	0.44	Bal.
AZ91–1.0Sm	9.12	0.17	0.55	0.95	Bal.
AZ91–1.5Sm	8.87	0.26	0.90	1.39	Bal.

**Table 2:** Fitted data of the Tafel curves. *E_corr_:* corrosion potentials, *I_corr_:* corrosion current density; *β_a_:* anode Tafel slopes; *β_c_:* cathode Tafel slopes; *R_p_:* polarization resistance.

Alloy	*E_corr_* (V_SCE_)	*I_corr_* (mA/cm^2^)	*β_a_* (mV/Dec)	*β_c_* (mV/Dec)	*R_p_* (Ω·cm^2^)
AZ91	−1.447	28.8	324.4	1062.1	3.8
AZ91–0.5Sm	−1.442	19.1	381.6	1574.5	7.0
AZ91–1.0Sm	−1.452	20.6	398.1	1325.4	6.5
AZ91–1.5Sm	−1.413	16.6	344.3	1681.1	7.5
